# 100 Million-year-old straight-jawed lacewing larvae with enormously inflated trunks represent the oldest cases of extreme physogastry in insects

**DOI:** 10.1038/s41598-022-16698-y

**Published:** 2022-07-26

**Authors:** Joachim T. Haug, Carolin Haug

**Affiliations:** 1 Biocenter, Ludwig-Maximilians-Universität München, Großhaderner Str. 2, 82152 Planegg-Martinsried, Germany; 2 GeoBio-Center at LMU, Richard-Wagner-Str. 10, 80333 Munich, Germany

**Keywords:** Zoology, Entomology, Palaeontology

## Abstract

Physogastry is a phenomenon occurring in Euarthropoda and describes an extreme inflation of (parts of) the trunk. It is best known from ticks, termite queens, or honey-pot ants, but can also be found in several other representatives of Euarthropoda. Physogastry has so far rarely been seen in the fossil record. We describe here an example of physogastry in two lacewing larvae (Neuroptera) enclosed in a single piece of Kachin amber (ca. 100 Ma old). We measured head and trunk ratios of different physogastric and non-physogastric representatives of Euarthropoda. Plotting these ratios shows that the new larvae, which display quite extremely inflated trunks, are very similar to ticks or honey-pot ants, but also to certain lacewing larvae of the group Berothidae (beaded lacewings). Outline analysis of head capsule and mouthparts (stylets) further suggests a position within Berothidae. Physogastry is presumed to be linked with living in confined spaces such as wood galleries or soil. Indeed, at least some larvae of Berothidae are known to live inside termite nests for part of their larval life phase, a habit the new larvae may also have had. The new record represents the oldest case of extreme physogastry in insects known to date.

## Introduction

Representatives of the group Euarthropoda—spiders, beetles, lobsters, centipedes and all their closer relatives—have to moult in order to grow. Indeed, their epidermis produces a chitinous cuticle, which is, basically, not expandable^1^. When moulting, the animal has in fact two layers of cuticle, an outer one that will be shed, and an inner one that is also already not truly expandable. In order to gain size during a moult, the inner cuticle is formed with distinct folds and is unfolded when the outer cuticle is moulted, allowing a size gain^2–4^.

**Figure 1:**
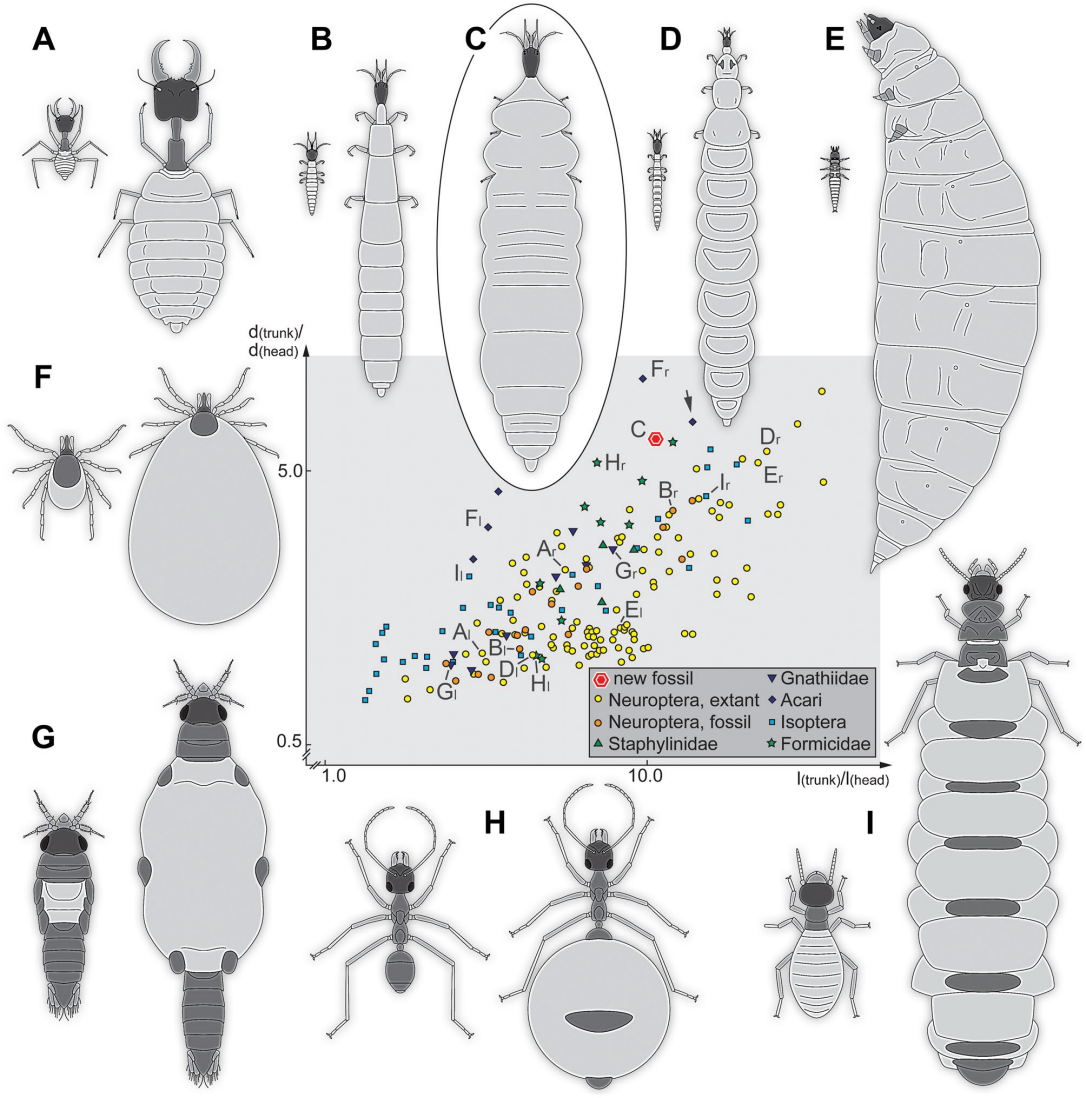
Examples of physogastric/inflated representatives of Euarthropoda and their non-physogastric/non-inflated counterparts and a scatter plot of body ratios (see Supplementary Table 1). The corresponding drawings of the same panel are always to the same scale; note the logarithmic scale; the small letters l and r behind the capital letters refer to the left and right specimen in the respective panel; the arrow marks the fossil physogastric tick reported by Peñalver et al.^17^. (**A–E**) Lacewing larvae. (**A**) Thread-winged lacewings (Crocinae), *Josandreva sazi*^18^, larva stage 1 (left) and 3 (right). (**B**) Fossils of possible beaded lacewings (Berothidae^19^), possible larva stage 1 (left, specimen 5835, CJW F 3198) and possible larva stage 3 (right, specimen 5833, CJW F 3197). (**C**) New fossil larva. (**D**) Beaded lacewings, *Lomamyia*^20^, larva stage 1 (left) and 3 (right). (**E**) Mantis lacewings (Mantispidae), *Mantispa uhleri*^21^, larva stage 1 (left) and 3 (right). (**F**) Ticks (Acari^9^), unfed female (left) and fed female (right). (**G**) Gnathiidae, *Gnathia africana*^22^, unfed zuphea (left) and fed praniza (right). (**H**) Honey-pot ant (Formicidae), *Myrmecocystus mexicanus* “normal” worker (left, bugguide #1588835) and worker in honey-pot state (right, bugguide #567398). (**I**) Termites (Isopoda), *Macrotermes gilvus*^13^, worker (left) and physogastric queen (right). *d(head)* diameter of head, *d(trunk)* diameter of trunk, *l(head)* length of head, *l(trunk)* length of trunk.

In this respect, a rare phenomenon in Euarthropoda seems remarkable, namely the extreme inflation of the trunk, or parts of it, often referred to as physogastry^5–8^. The best-known example of extreme inflation is that of ticks: when feeding blood on the host their posterior trunk (hysterosoma) inflates to several times its original size (Fig. 1F)^9–11^. Other well-known extreme cases are the inflated posterior trunks (abdomen, gaster) of termite queens (Fig. 1I)^12–16^ and honey-pot ants (Fig. 1H)^6^.

Besides these, there are also less well-known cases of physogastry. Other mites can expand in a fashion similar to that of ticks^5,7^, but are in fact more comparable to termite queens, as inflation is related to reproduction. Other queens, for example, among ants^23–26^ and bees^27,28^, also show this feature, as do beetles and flies imitating termites and ants (adults^29–33^; larvae^33–35^). Some beetles also show physogastry related to enlarged ovaries^36,37^.

More rarely, the trunk can expand in the anterior region only, as in some beetles that have a relation to termites^38^, and in some isopodan crustaceans (Gnathiidae; Fig. 1G). The latter expand enormously in the larval stages, when feeding on the blood of fish, but also as adult females when carrying their brood^22,39–42^; in rare cases the phenomenon has been reported in immatures males^43^. Another lesser-known case of physogastry is that of certain lacewing larvae displaying straight jaws being representatives of the groups Dilaridae (pleasing lacewings^20,44^) and Berothidae (beaded lacewings; Fig. 1D;^20^ [^45^, their p. 203]). In all cases of physogastry, we need to assume that the cuticle of these animals has special folds, or other structural peculiarities, to allow the extreme change in volume when feeding (e.g., in ticks, honey-pot ants, caterpillars, presumably lacewings^46–48^) or growing oversized gonads (e.g., in termites, ants, beetles^49^).

The patchy distribution of physogastry on the phylogenetic tree of Euarthropoda indicates that this ability evolved several times independently (e.g.,^30^). Fossils can be very informative for reconstructing the evolutionary history of these occurrences, not least by providing minimum ages for them. So far, clear-cut cases of physogastry have not been explicitly reported from the fossil record. We herein report lacewing larvae preserved in ca. 100 million-year-old Kachin amber (Myanmar) displaying extremely enlarged trunks.

## Results

### Description of the amber piece

**Figure 2:**
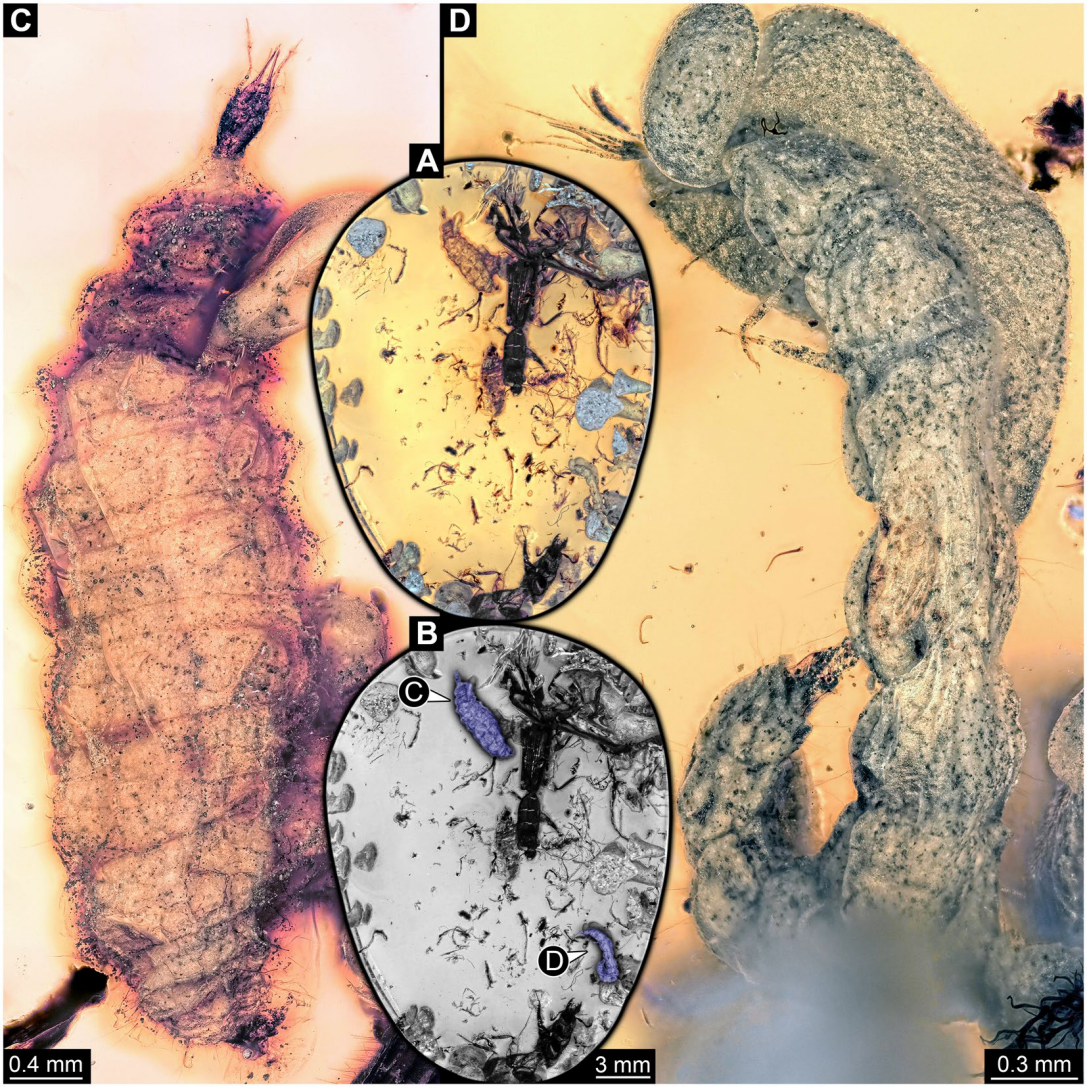
PED 1794, Kachin amber. (**A**) Overview of amber piece. (**B**) Desaturated version of **A** with the two lacewing larvae colour-marked. (**C**) Lacewing larva 1, dorsal view. (**D**) Lacewing larva 2, dorso-lateral view.

The amber piece (PED 1794) includes several inclusions (Fig. 2A,B). Of central interest are two larvae interpreted as lacewings (see further below for identification). Syn-inclusions include a small immature cockroach and two adult beetles.

**Figure 3:**
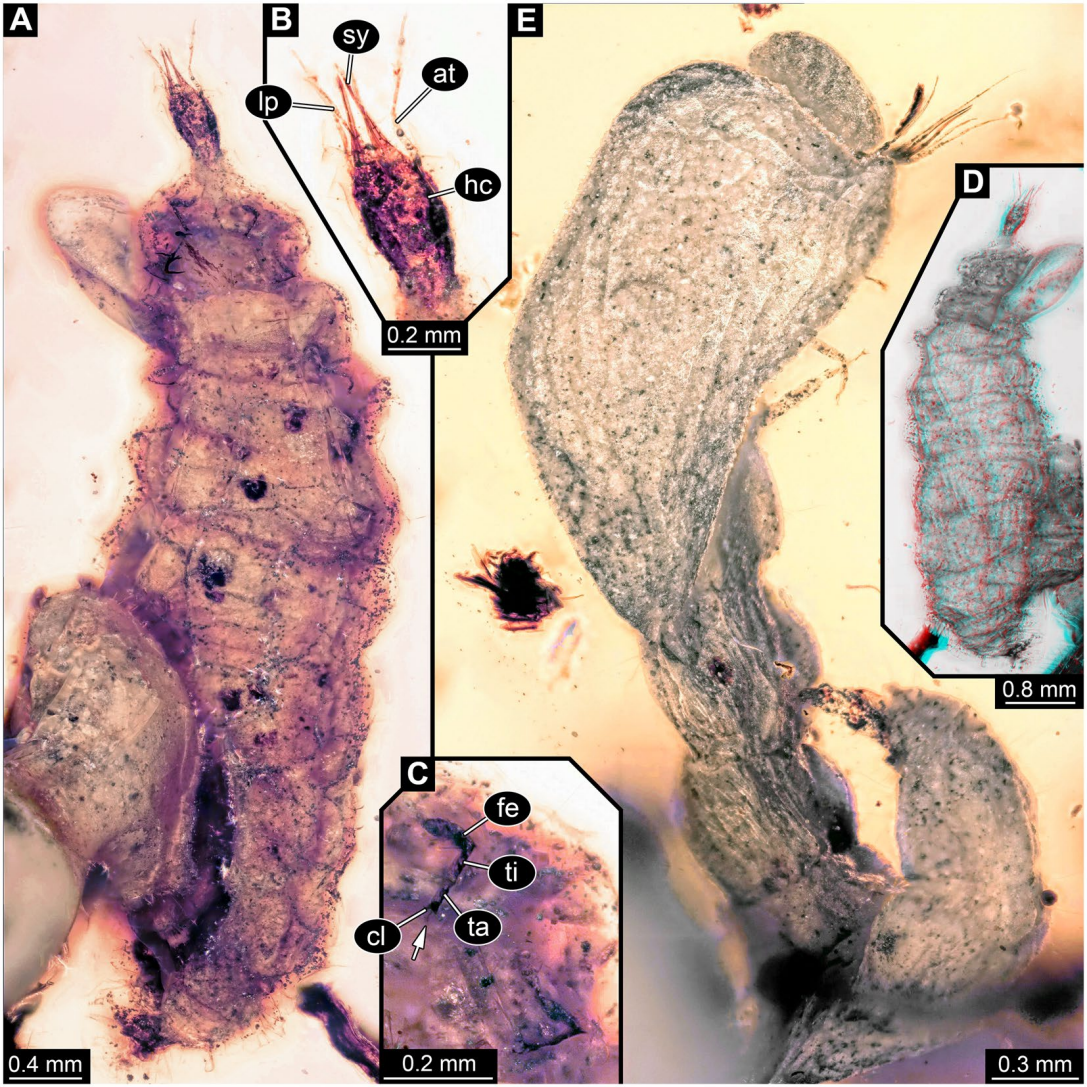
PED 1794, Kachin amber, continued. (**A–C**) Lacewing larva 1, ventral view. (**A**) Overview. (**B**) Detail of head. (**C**) Detail of thorax appendage with empodium (arrow). (**D**) Lacewing larva 1, dorsal view, red-cyan stereo anaglyph (use red-cyan glasses for the stereo effect). (**E**) Lacewing larva 2, presumed ventro-lateral view, largely concealed by bubbles. *cl* claw, *fe* femur, *ta* tarsus, *ti* tibia.

*Larva 1* well exposed in both dorsal and ventral views (Figs. 2C, 3A–D); body organised into small head and very large trunk.

*Head* capsule rectangular to oval in both dorsal and ventral views (Figs. 2C, 3A,B), widest in the middle, narrower anteriorly and posteriorly, longer than wide (1.5x); details of eyes not apparent; appendage of post-ocular segment 1 (antenna) long and slender, about as long as head capsule, with at least four elements, but joints not well apparent; no external structures of post-ocular segment 2 apparent (intercalary segment); appendages of post-ocular segments 3 and 4 (mandible and maxilla) forming straight stylets (Fig. 3B), shorter than head capsule, strongly tapering towards their anterior tip; distal parts of appendages of post-ocular segment 5 (labial palp) well apparent, elongate, slightly longer than stylets, shorter than antenna (Fig. 3B), with subdivision into elements not well apparent.

*Trunk* much larger than head, significantly longer (more than 11x), wider than head (more than 6 × at widest point; Figs. 2C, 3A); exact subdivision into segments unclear, but three anterior segments (thorax) well apparent, and nine posterior segments (abdomen) probably present; no distinct set-off neck region between head and trunk.

*Thorax* segment 1 (prothorax) trapezoid in dorsal view, much wider posteriorly than anteriorly, segments 2 and 3 (meso- and metathorax) slightly widening posteriorly; each segment with a pair of ventral locomotory appendages (legs; Fig. 3A), sub-similar, about as long as segments, inserting far laterally; proximal region of legs not well apparent, subdivision into three major parts, femur, tibia and tarsus distinguishable (Fig. 3C); tarsus with a pair of distal claws and a median trumpet-shaped empodium (white arrow in Fig. 3C).

*Abdomen* segments appear overall soft and seem to lack sclerites; anterior six(?) segments more or less rectangular in dorsal view, of similar length, slightly wider than thorax segments; posterior segments tapering posteriorly.

*Larva 2* not as well exposed as larva 1 (Figs. 2D, 3E); overall morphology very similar to that of larva 1, including size, yet fewer details available. Body preserved less straight, partly twisted, observable mostly in lateral aspect; anterior body region covered from one side by granulated bubble, hindering observation from this orientation.

### Quantitative comparison to other lacewing larvae

When plotting ratios of head-to-trunk dimensions of a selection of Euarthropoda (with a main focus on lacewings) (Supplementary Fig. 1; see also Supplementary Table 1, Supplementary Text 1), extant larvae of three major lacewing groups, namely Berothidae, Dilaridae, and Mantispidae, occupy positions in the upper right area of the plot, indicating oversized trunk regions. Some specimens of Ithonidae and Hemerobiidae also plot in this area. Larvae of Mantispidae occupy the most extreme positions. Among the fossil lacewing larvae, some exhibit a rather large trunk, resulting in a far-right position. Some specimens plot even further to the right than the new larva 1 (four cases of putative larvae of Osmylidae and Berothidae), but the new larva plots further up due to the very broad trunk. So far known fossil larvae tend to have more slender trunks.

### Quantitative comparison to other cases of physogastry

Comparing the cases of Dilaridae, Berothidae and Mantispidae with other representatives of Euarthropoda reveals that similarly extreme cases occur in termites (Isoptera; Fig. 1I), ants (Formicidae; Fig. 1H) and ticks (Acari; Fig. 1F). Blood-feeding isopodan crustaceans (Gnathiidae; Fig. 1G) can also inflate drastically, but do not reach extreme proportions. The same applies to termitophilous rove beetles (Staphylinidae; Fig. 1).

### Quantitative comparison of head and stylet shape

The SHAPE analysis of head and stylet outline (see Supplementary Table 2 and Supplementary Text 1 for details on the specimens) resulted in only two principal components (for the results of the principal component analysis, see Supplementary Text 2, Supplementary Files 1, 2). PC1 explains 95.4% of the entire variation and is dominated by the relative length of the stylets. Low values indicate shorter stylets, high values indicate longer stylets. PC2 explains 2.0% of the entire variation and is dominated by the shape of the anterior rim of the head capsule. Low values indicate convex anterior rims, high values indicate concave anterior rims.

Extant larvae of Berothidae and Dilaridae do not strongly separate (Supplementary Fig. 2). However there is a certain area in PC1 (and PC2) occupied only by larvae of Berothidae (i.e. where larvae of Dilaridae do not plot). There are some fossil larvae with straight stylets that plot in this particular area. This is also the case in the new fossil larva (larva 1). The fossil larvae with straight stylets largely overlap with the range of the extant larvae.

## Discussion

### Identity of the new fossils

Although the new fossils may appear alienating at first, the forward-projecting mouthparts forming stylets and the presence of trumpet-shaped empodia on the legs immediately identify them as larvae of Neuroptera. The overall head shape, with straight stylets, further indicates that the specimens are representatives of Dilaridae or Berothidae^19^. Both groups seem to be not closer related according to recent phylogenetic reconstructions^50,51^. Straight stylets are therefore apomorphic for the group Dilaridae as well as another unnamed group, which includes also Berothidae^51^. Considering the comparatively short stylets, an interpretation as larvae of Berothidae seems more likely, which is supported by the quantitative morphological analysis (Supplementary Fig. 2). The new fossils differ from extant known cases with large trunks by having relatively broader trunks. In extant larvae, the trunks in beaded lacewings (Berothidae) are broader than those of pleasing lacewings (Dilaridae), further suggesting that the new specimens are representatives of Berothidae.

Known larvae of Berothidae from Kachin amber either have non-inflated trunks (Fig. 1B left) or partly inflated ones (Fig. 1B right). Together with the two new fossils (Fig. 1C), it seems likely that the three conditions (without inflated trunk, with partly inflated trunk, with extremely inflated trunk) represent three larval stages, either of a single fossil species or of several closely related ones. We therefore see it as the most likely interpretation that the two new fossils are stage 3 larvae of beaded lacewings.

### The phenomenon of physogastry: setting a comparative frame

The term physogastry has been used in different instances in the literature. Mergelsberg^8^ used it specifically for cases in which the inflation is due to enlarged reproductive organs. Here we use the term in the wider sense, referring to any extreme inflation of the trunk region (as in [^52^, his p. 16]). This use is better suited for discussing fossil cases, for which the possible causes can only be inferred indirectly. The case in Gnathiidae demonstrates that the results of enlarged trunks due to feeding or reproductive organs are very similar. Also, a differentiation of inflation of anterior and posterior trunk (e.g.,^38^) is likely to complicate the situation rather than enlightening it. Hence, we use physogastry also for both cases.

It is also relevant to consider which “normal” conditions possible cases of physogastry should be compared to. In most cases, it seems most practical to contrast individuals of the same developmental stage, such as workers in honey-pot ants (Fig. 1H), or unfed ticks (Fig. 1F). In other cases, it may be necessary to consider other morphs of the same species, as in termites (Fig. 1I). In the case of lacewing larvae (Fig. 1A–E), non-inflated and inflated individuals will belong to different larval stages, with non-inflated ones representing stage 1 larvae, while inflated ones most often represent stage 3 larvae. The comparison might therefore appear at first more extreme, yet this is caused by the fact that two moults occurred between the two stages, while in the case of honey-pot ants, for example, no moulting is involved. Also, comparisons over wider phylogenetic areas is important for further discussions (see discussion in^53^). Another aspect is how noticeable physogastric individuals can be throughout different groups of Euarthropoda. In general, physogastry becomes apparent as such due to a very large-sized trunk in comparison to the head. Indeed, simple head vs. trunk ratios (width and diameter) provided a plot (Fig. 1), the upper right area of which mostly includes individuals that have traditionally been considered to be physogastric such as late stage larvae of Berothidae (Fig. 1B, D), but especially termite queens (Fig. 1I). The new fossil (Fig. 1C) plots a bit off from the few known examples of physogastric lacewing larvae from the extant fauna, but clearly is among other individuals that appear physogastric. We therefore consider the new fossils as clear cases of physogastry.

### Physogastry in holometabolan larvae and lacewings

We assume that cases of physogastry are probably underestimated among holometabolan larvae. Many grubs and caterpillars in their later stages factually have very long and broad trunks and are rather soft, which would then qualify for physogastry as delineated above.

The larvae of the group Holometabola (including beetles, bees, butterflies, flies, but also lacewings) are in principle 'eating machines'^54,55^. It is therefore not surprising that some evolved the ability to expand the trunk volume quite dramatically. Factually, many holometabolan larvae, including different lacewings, show a tendency towards physogastry (e.g., Psychopsidae^56^; Osmylidae^57^) Yet, extreme cases in lacewings, in which the term physogastry has been applied, are those of later stage larvae of pleasing lacewings (Dilaridae^58^) and beaded lacewings (Berothidae^20^). Surprisingly, the term has not been applied for later larvae of mantis lacewings (Mantispidae), although our comparison demonstrates the trunk is comparatively even more dramatically inflated (Fig. 1E). In fact, in the comparative frame used herein mantis lacewing larvae are more extreme than, for example, termite queens. Most likely, the mantis lacewing case is more extreme due to the fact that even the thorax segments are inflated. Indeed, in contrast, in most other cases of physogastry, the anterior trunk segments (for example, in Gnathiidae, termites, or ants) remain largely unaffected (Fig. 1G–I). Extant larvae of Berothidae and Dilaridae, as well as the new fossil cases, display enlarged anterior trunk segments as well, which seems to represent a special case among these lacewing larvae.

### Physogastry in the fossil record

So far, clear-cut cases of physogastry seem to not have been explicitly reported from the fossil record. Still, it is worth to consider some of the candidate groups. Termites are present already in the Cretaceous^59^, yet there is so far no known case of a physogastric queen. It is not totally surprising, given that not all termite queens exhibit extreme physogastry^60^. Indeed, the phenomenon appears to be especially expressed in termites specialised in foraging^61^, but not in wood-dwelling ones. During the Cretaceous, termites were likely wood-dwelling forms, and physogastric termite queens might have not evolved yet. Similarly, highly specialised ants are well known in the Cretaceous^62^, but no case of physogastric queens has ever been reported.

Few lineages of Diptera also exhibit physogastry as adults in relation to blood-feeding, e.g., bird flies^63^. While there are fossil representatives of some of these lineages, none of them has shown clear-cut signs of physogastry^63^.

Fossil ticks are still rare, and most specimens known so far are non-inflated individuals^64,65^; none of them has been referred to by the term 'physogastric'. Yet, inflated ticks have been reported by Shi et al.^66^ and Poinar^67^. Most recently, Peñalver et al.^17^ reported ticks on dinosaurs, including a female inflated specimen. This specimen plots in the 'physogastric area' (arrow in Fig. 1), with an even slightly wider and longer trunk than in the new fossils.

Possible relatives of Gnathiidae have been reported from the Jurassic^68^. Several such fossils (of the group *Urda*) are, for example, known from the Solnhofen Lithographic Limestones [^68^, their Table 1], yet none of these fossils has been reported to show inflation of the trunk region.

Some already reported fossil larvae of the group Berothidae have a moderately enlarged trunk, most likely representing stage 2 larvae. These specimens include fossils from the Cretaceous [^19^, their Figs. 6, 12] as well as from the Eocene [^19^, their Fig. 2] [^69^, their Figs. 8–11].

It appears that most lineages nowadays known to exhibit physogastry lack a clear case in the fossil record, at least in the Cretaceous. The ticks reported by Shi et al.^66^ and Peñalver et al.^17^ seem to be the only exceptions, yet without using the term physogastry. The new fossil larvae therefore represent the only second case of extreme physogastry in the Cretaceous and the oldest one for the group Insecta.

### Function of physogastry and life style of the new fossils

The function of physogastry in lacewing larvae is still difficult to appreciate, even in the extant representatives. Most lacewing larvae are predators, a lifestyle difficult to reconcile with a strongly enlarged trunk. In other cases, physogastry is clearly coupled to a reduced or almost absent mobility^6,70^.

For pleasing lacewing larvae, a life style in wood galleries of other holometabolan larvae^20,58,71^ and soil^58,72^ was inferred. Beaded lacewing larvae live in termite nests, mantis lacewings larvae in egg sacs of spiders or in nests of eusocial hymenopterans (wasps, bees^73^, their p. 103).

Badano et al.^58^ suggested that the 'enlarged trunk' morphology may be functionally coupled to life in a confined space. In larvae of checker beetles, living either in bee nests or in wood galleries of other holometabolan larvae, also an inflation of the trunk, in later larval stages, is well apparent^74^. A similar phenomenon is found in certain larvae of ground beetles living in soil as parasites of leaf beetle immatures^75^. Living with termites also seems to be coupled to a certain degree to physogastry, as is the case for some beetles and flies^8,76^.

It is not fully clear whether fossil larvae of beaded lacewings also lived in termite nests (see discussion in^69^), but it seems likely to have been the case. Fossils reported herein are not preserved together with termites (but with a cockroach and beetles), indicating that these specimens were outside a termite nest. A point to consider is that mantis lacewings living in bee nests will be killed by bee workers if they moult to the adult stage inside the nest [^73^, their p. 103]. It can then be expected that beaded lacewings living in termite nests would face the same situation, and would have to leave the nest at a certain time in their ontogeny. Gurney^20^ discussed whether extremely physogastric beaded lacewing larvae could be close to pupation, but remained inconclusive. Cocoons of beaded lacewings have been found under loose bark or even outside wood, under a log or branch^77^. This indicates that the late stage larvae leave the termite nest at a certain point. It can then be legitimately speculated that the new fossil larvae indeed originally lived in a termite nest, but left it in order to pupate outside.

## Material and methods

### Material

The single amber piece which is the focus of this study is from Kachin amber, Myanmar, and therefore considered of Cretaceous age, about 100 million years^66,78,79^. The specimen was legally purchased via the online platform ebay.com from the trader burmite-researcher. The specimen is now deposited in the Palaeo-Evo-Devo Research Group Collection of Arthropods, Ludwig-Maximilians-Universität München, Germany, under repository number PED 1794.

### Documentation methods

The amber piece was documented using a Keyence VHX-6000 digital microscope with standard procedures. Composite imaging was performed as well as HDR. Different backgrounds and light settings (polarised vs. non-polarised light) were used to find the combination providing the best contrast (for details, see^80^ and references therein).

### Measurements

For comparison of the new specimens with other physogastric representatives of Euarthropoda as well as their non-physogastric counterparts, we performed several measurements based on illustrations in the literature and in the database bugguide.net (for details, see Supplementary Table 1 and Supplementary Text 1). Our measurements included the length and diameter of the anterior body region and of the remaining body. The anterior body region corresponds to the head capsule for representatives of Insecta, the cephalothoracic shield in representatives of Isopoda, as well as the gnathosoma in representatives of Acari. Hence, this region basically represents the “functional head” in all cases. Forward-projecting mouthparts, antennae, and other similar structures were not included. The remaining part of the body represents the trunk. However, larva 2 discussed in this paper could not be measured reliably.

### Quantitative morphology via elliptic fourier transformation

We re-used parts of data of the head and stylet shape from Haug et al.^19^. Specimens included are extant larvae of the groups Berothidae and Dilaridae as well as fossil larvae potentially representing the same two groups. Additionally, of two specimens depicted in Badano et al.^58^ the outlines of the head capsule and stylets were redrawn in Adobe Illustrator CS2. Finally, of one of the newly reported larvae the outline of head capsule and stylets were also redrawn and included into the data set. All these outline drawings were transformed into bitmap files and included into an Elliptic Fourier Analysis (EFA) performed with the software SHAPE^81^, followed by a principal component analysis (PCA) (for details, see^19,82^).

## Supplementary Information

Supplementary Legends.

Supplementary Information 1.

Supplementary Information 2.

Supplementary Information 3.

Supplementary Information 4.

Supplementary Information 5.

Supplementary Information 6.

Supplementary Information 7.

Supplementary Information 8.

## Data Availability

All data is included in this paper.
