# Supplementary material for: 100 Million-year-old straight-jawed lacewing larvae with enormously inflated trunks represent the oldest cases of extreme physogastry in insects
Source: Sci Rep. 2022 Jul 26;12:12760. doi: 10.1038/s41598-022-16698-y (PMC9325756; doi:10.1038/s41598-022-16698-y)
Supplement: Supplementary file 1 — Supplementary Legends. [file 41598_2022_16698_MOESM1_ESM.docx]

**Suppl.-Fig. 1.** Scatter plot of body ratios of the new fossil (larva 1) and further lacewing larvae, both extant and fossil, as well as of a selection of non-lacewing representatives of Euarthropoda (see Suppl.-Tab. 1); same scatter plot as in Fig. 1, but with lacewing groups differentiated, and without differentiation of non-lacewing groups; note the logarithmic scale. *d(head)* diameter of head, *d(trunk)* diameter of trunk, *l(head)* length of head, *l(trunk)* length of trunk.

**Suppl.-Fig. 2.** Plotted values of PC1 and PC2 for extant larvae of Berothidae and Dilaridae, for fossil lacewing larvae with straight stylets resembling Berothidae and/or Dilaridae, and for the new fossil (larva 1).

**Suppl.-Tab. 1.** Detailed information about the specimens measured for the ratios used in the scatter plot of Fig. 1. *d(head)* diameter of head, *d(trunk)* diameter of trunk, *l(head)* length of head, *l(trunk)* length of trunk.

**Suppl.-Tab. 2.** Detailed information about the specimens included into the shape analysis. *Eoc* Eocene, *K* Cretaceous, *Mio* Miocene.

**Suppl.-Text 1.** References from Suppl.-Tabs. 1 and 2 not referred to in the main text.

**Suppl.-Text 2.** Results of the principal component analysis.

**Suppl.-File 1.** Graphical representation of the factor loadings of the principal component analysis.

**Suppl.-File 2.** Files resulting from the shape analysis, including chain codes, aligned shapes, and principal component analysis.
