# Supplementary figures and images for: 100 Million-year-old straight-jawed lacewing larvae with enormously inflated trunks represent the oldest cases of extreme physogastry in insects
Source: Sci Rep. 2022 Jul 26;12:12760. doi: 10.1038/s41598-022-16698-y (PMC9325756; doi:10.1038/s41598-022-16698-y)

-2S.D.

Mean

+2S.D.

PC1

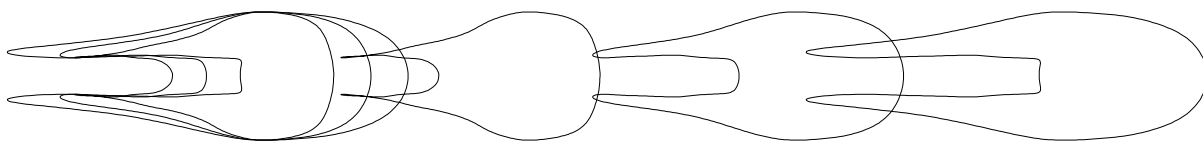

PC2

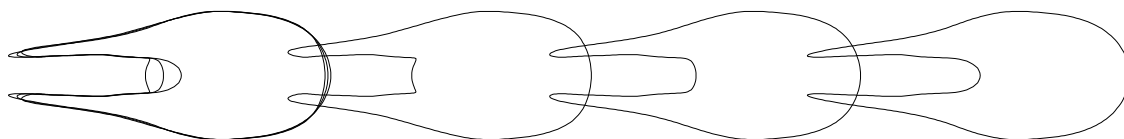

Supplement: Supplementary file 2 — Supplementary Information 1. [file 41598_2022_16698_MOESM2_ESM.pdf]

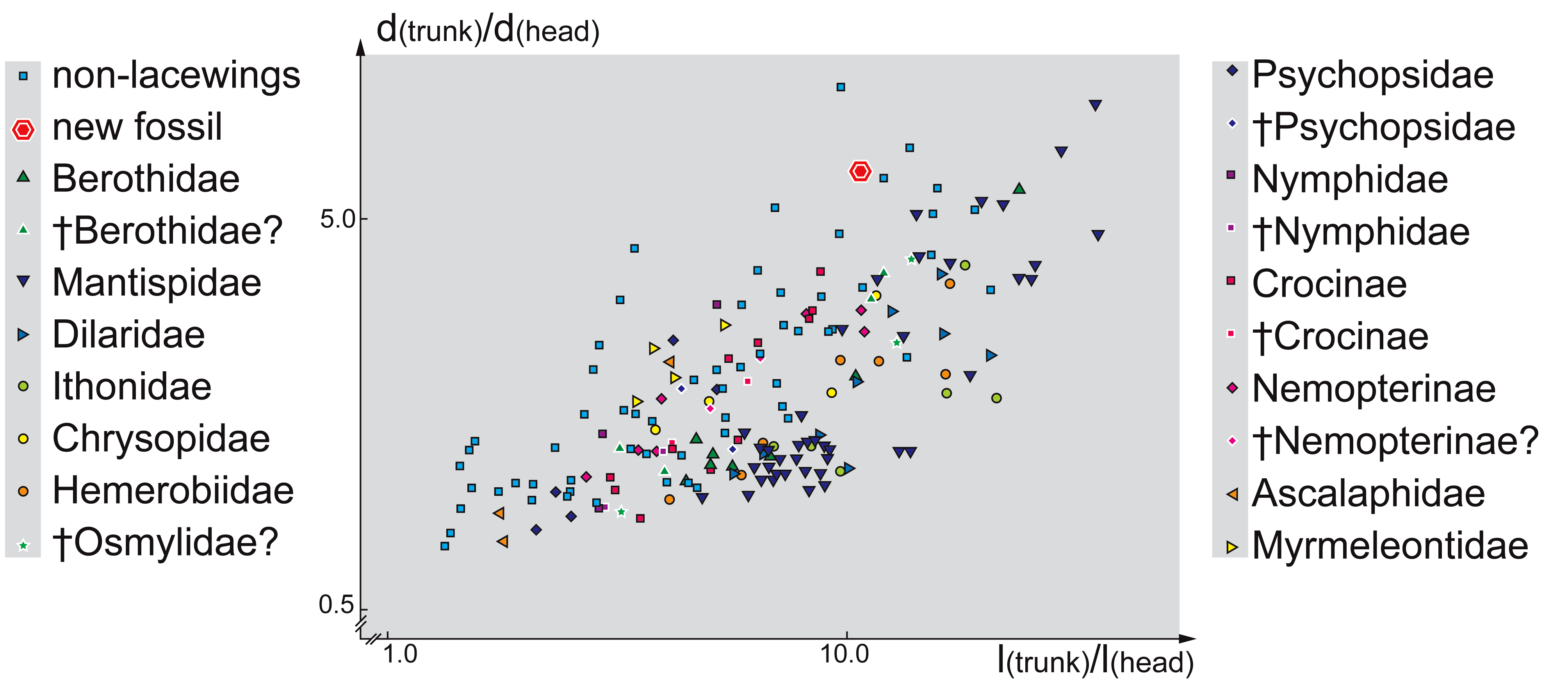

Supplement: Supplementary file 8 — Supplementary Information 7. [file 41598_2022_16698_MOESM8_ESM.tif]

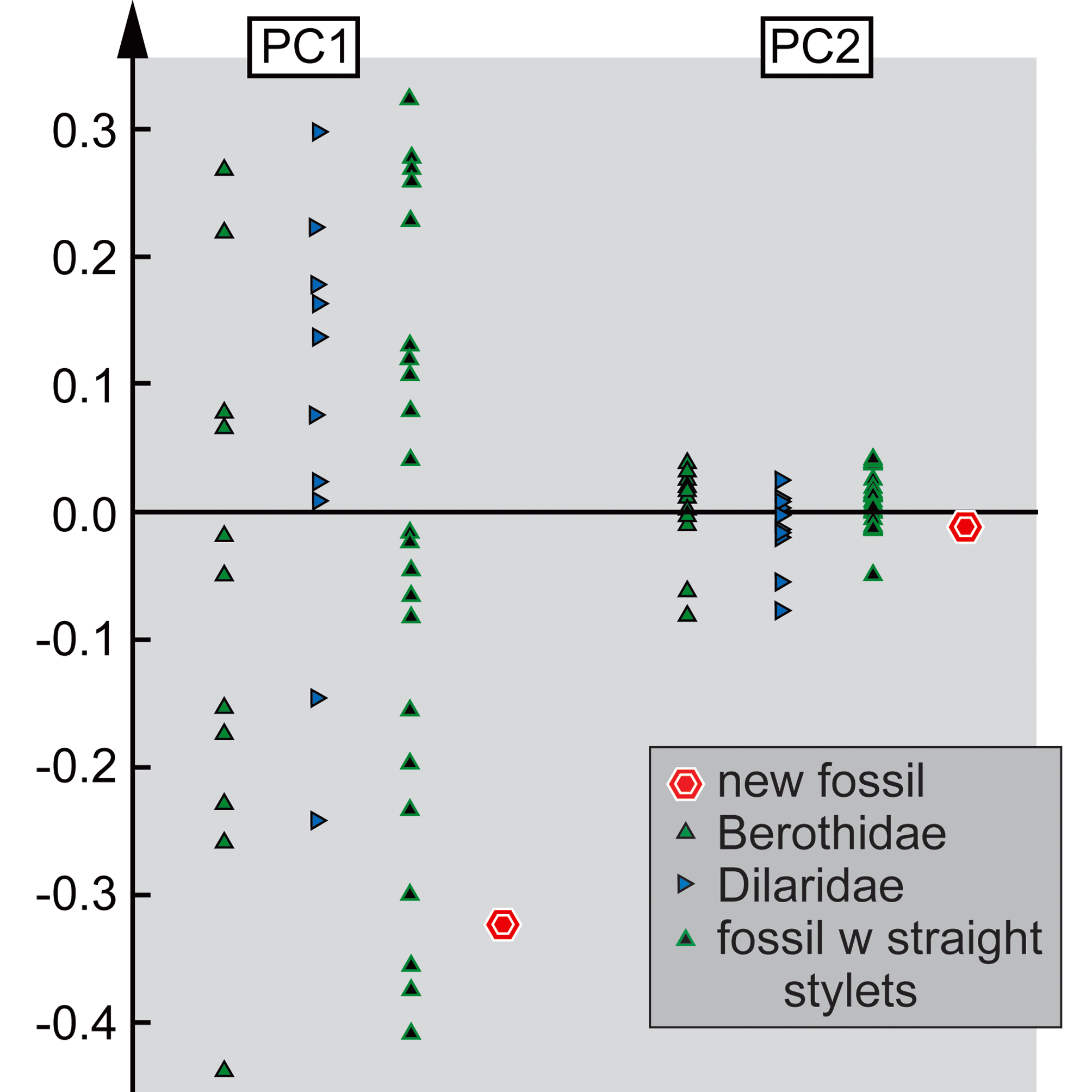

Supplement: Supplementary file 9 — Supplementary Information 8. [file 41598_2022_16698_MOESM9_ESM.tif]
