# Supplementary material for: 100 Million-year-old straight-jawed lacewing larvae with enormously inflated trunks represent the oldest cases of extreme physogastry in insects
Source: Sci Rep. 2022 Jul 26;12:12760. doi: 10.1038/s41598-022-16698-y (PMC9325756; doi:10.1038/s41598-022-16698-y)
Supplement: Supplementary file 6 — Supplementary Information 5. [file 41598_2022_16698_MOESM6_ESM.doc]

**Additional references for Supplementary Tables 1 and 2:**

Adams, P.A. 1959. Neuroptera: Myrmeleontidae and Chrysopidae. Insects of Micronesia 8, 13–33.

Anonymus 1975. A parasite and a spider. Nasionale Museum Nuus / National Museum News (Culna) 1975, 1 (no pagination).

Aspöck, U. & Aspöck, H. 1999. Kamelhälse, Schlammfliegen, Ameisenlöwen. Wer sind sie? (Insecta: Neuropterida: Raphidioptera, Megaloptera, Neuroptera). Stapfia 60(138), 1–34.

Aspöck, U. & Aspöck, H. 2007. Verbliebene Vielfalt vergangener Blüte. Zur Evolution, Phylogenie und Biodiversität der Neuropterida (Insecta: Endopterygota). Denisia 20, 451–516.

Bissett, J.L. & Moran, V.C. 1967. The life history and cocoon spinning behaviour of a South African mantispid (Neuroptera: Mantispidae). Journal of the Entomological Society of southern Africa 30, 82–95.

Brauer, F. 1852. Verwandlungsgeschichte der *Mantispa pagana*. Archiv für Naturgeschichte 18, 1–2.

Brauer, F. 1855. Beiträge zur Kenntniss des inneren Baues und der Verwandlung der Neuropteren. Verhandlungen des Zoologisch-Botanischen Vereins in Wien 5,

701–726.

Brauer, F. 1869. Beschreibung der Verwandlungsgeschichte der *Mantispa styriaca* Poda und Betrachtungen über die sogenannte Hypermetamorphose Fabre′s. Verhandlungen der Kaiserlich-Königlichen Zoologisch-Botanischen Gesellschaft in Wien 19, 831–840.

Davidson, J.A. 1969. Rearing *Mantispa viridis* Walker in the laboratory (Neuroptera, Mantispidae). Entomological News 80, 29–31.

Dobosz, R. & Górski, G. 2008. New data on *Nyrma kervilea* (Neuroptera: Berothidae). In: Devetak, D. & Klenovšek, T. (eds.), Tenth International Symposium on Neuropterology, 22–25 June 2008, Piran, Slovenia. Abstract book. Faculty of Natural Sciences and Mathematics, Maribor, pp. 33.

Engel, M.S. 2016. Two new genera of Cretaceous dustywings in amber from northern Myanmar (Neuroptera: Coniopterygidae). Novitates Paleoentomologicae 17, 1–16.

Engel, M.S. & Grimaldi, D.A. 2008. Diverse Neuropterida in Cretaceous amber, with particular reference to the paleofauna of Myanmar (Insecta). Nova Supplementa Entomologica, Keltern 20, 1‒86.

Froggatt, W.W. 1907. Australian Insects. William Brooks and Company, Ltd., Sydney.

Ghilarov, M.S. 1962. Личинка *Dilar turcicus* Hag. и положение семейства Dilaridae в отряде сетчатокрылых (Planippenia) [=The larva of *Dilar turcicus* Hag. and the position of the family Dilaridae in the order Planipennia]. Entomological Review [=Энтомологическое Обозрение; =Entomologicheskoe Obozrenie] 41, 402–416.

Ghosh, C.C. 1910. XXVII. Entomological notes. *Croce filipennis*, Westw. Journal of the Bombay Natural History Society 20, 530–532.

Grebennikov, V.V. 2004. Grub-like larvae of Neuroptera (Insecta): a morphological review of the families Ithonidae and Polystoechotidae and a description of *Oliarces clara*. European Journal of Entomology 101, 409–418.

Haug, G.T., Haug, C., Pazinato, P.G., Braig, F., Perrichot, V., Gröhn, C., Müller, P. & Haug, J.T. 2020. The decline of silky lacewings and morphological diversity of long-nosed antlion larvae through time. Palaeontologia Electronica 23(2), a39.

Haug, G.T., Baranov, V., Wizen, G., Pazinato, P G., Müller, P., Haug, C. & Haug, J.T. 2021b. The morphological diversity of long-necked lacewing larvae (Neuroptera: Myrmeleontiformia). Bulletin of Geosciences 96, 431–457.

Haug, G.T., Haug, C. & Haug J.T. 2021c. The morphological diversity of spoon-winged lacewing larvae and the first possible fossils from 99 million-year-old Kachin amber, Myanmar. Palaeodiversity 14, 133–152.

Haug, G.T., Haug, C., van der Wal, S., Müller, P. & Haug, J.T. 2022. Split-footed lacewings declined over time: indications from the morphological diversity of their antlion-like larvae. PalZ 96, 29–50.

Henry, C.S. 1976. Some aspects of the external morphology of larval owlflies (Neuroptera: Ascalaphidae), with particular reference to *Ululodes* and *Ascalopterynx*. Psyche 83(1), 1–31.

Hölldobler, B. & Wilson, E.O. 1990. The Ants. Harvard University Press, Cambridge.

Hölzel, H. 1999. Die Nemopteriden (Fadenhafte) Arabiens. Stapfia 60, 129–146.

Hoffman, K.M., & Brushwein, J.R. 1992. Descriptions of the larvae and pupae of some North American Mantispinae (Neuroptera: Mantispidae) and development of a system of larval chaetotaxy for Neuroptera. Transactions of the American Entomological Society 118, 159–196.

Hungerford, H.B. 1936. The Mantispidae of the Douglas Lake, Michigan Region, with some biological observations (Neurop.). Entomological News 47, 85–88.

Jandausch, K., Pohl, H., Aspöck, U., Winterton, S.L. & Beutel, R.G. 2018. Morphology of the primary larva of *Mantispa aphavexelte* Aspöck & Aspöck, 1994 (Neuroptera: Mantispidae) and phylogenetic implications to the order of Neuroptera. Arthropod Systematics & Phylogeny 76, 529–560.

Janzen, J.W. 2002. Arthropods in Baltic amber. Ampyx Verlag, Halle, 167 pp.

Kuroko, H. 1961. On the eggs and first-instar larvae of two species of Mantispidae. Esakia 3, 25–32.

Liu, X.-Y., Aspöck, H., Winterton, S.L., Zhang, W.-W. & Aspöck, U. 2017. Phylogeny of pleasing lacewings (Neuroptera: Dilaridae) with a revised generic classification and description of a new subfamily. Systematic Entomology 42, 448–471.

Lucchese, E. 1955. Ricerche sulla *Mantispa perla* Pallas (Neuroptera Planipennia—Fam. Mantispidae). I Nota preventiva su nuovi reperti concernenti l′etologia della larva della 1a età. Annali della Facoltà di Agraria della Università degli Studi di Perugia 11, 242–262.

MacLeod, E.G. 1960. The immature stages of *Boriomyia fidelis* (Banks) with taxonomic notes on the affinities of the genus *Boriomyia* (Neuroptera: Hemerobiidae). Psyche: A Journal of Entomology 67, 26–40.

MacLeod, E.G. 1964. A comparative morphological study of the head capsule and cervix of larval Neuroptera (Insecta). Ph.D. dissertation. Harvard University, Cambridge, Massachusetts, USA, 528 pp.

McKeown, K.C. & Mincham, V.H. 1948. The biology of an Australian mantispid (*Mantispa vittata* Guérin). Australian Zoologist 11, 207–224.

Mehra, B. 1965. Biology of *Chrysopa madestes* Banks (Neuroptera, Chrysopidae). Indian Journal of Entomology 27, 398–407.

Merti, C. 1940. Contribucion al estudio de *Mantispa decorata* Erd. (Hemip. Cor.). Revista de la Sociedad Entomológica Argentina 10, 304–307.

Minter, L.R. 1990. A comparison of the eggs and first-instar larvae of *Mucroberotha vesicaria* Tjeder with those of other species in the families Berothidae and Mantispidae (Insecta: Neuroptera). In: Mansell, M.W. & Aspöck, H. (eds.), Proceedings of the Third International Symposium on Neuropterology, Kruger National Park, South Africa, 3–4 February 1988; South African Department of Agricultural Development, Pretoria, South Africa, pp. 115–129.

Minter, L.R. 1992. The egg and larval stages of *Nallachius krooni* Minter (Insecta: Neuroptera: Dilaridae). In: Canard, M., Aspöck, H. & Mansell, M.W. (eds.), Current Research in Neuropterology, Proceedings of the Fourth International Symposium on Neuropterology, Bagnères-de-Luchon, France, 24–27 June 1991; Privately printed, Toulouse, France, pp. 261–269.

Möller, A., Minter, L.R. & Olivier, P.A.S. 2006. Larval morphology of *Podallea vasseana* Navás and *Podallea manselli* Aspöck & Aspöck from South Africa (Neuroptera: Berothidae). African Entomology 14, 1–12.

Monserrat, V.J. 1983b. *Pterocroce capillaris* (Klug, 1836) en Europa (Neur., Plan., Nemopteridae). Neuroptera International 2, 109–128.

Monserrat, V.J. 1988. Revisión de los diláridos ibéricos (Neuropteroidea, Planipennia: Dilaridae). EOS Revista Española de Entomología 64, 175–205.

Monserrat, V.J. 1996. Larval stages of European Nemopterinae, with systematic considerations on the family Nemopteridae (Insecta, Neuroptera). Deutsche Entomologische Zeitschrift, Neue Folge, 43, 99–121.

Monserrat, V.J. 2006. Nuevos datos sobre algunas especies de la familia Berothidae (Insecta: Neuroptera). Heteropterus Revista de Entomología 6, 173–207.

Monserrat, V.J. 2008. Nuevos datos sobre algunas especies de Nemopteridae y Crocidae (Insecta: Neuroptera). Heteropterus Revista de Entomología 8, 1–33.

Monserrat, V.J. & Díaz-Aranda, L.M. 1989. Estadios larvarios de los Neuropteros Ibericos. V: *Mantispa styriaca* (Poda, 1761) (Planipennia: Mantispidae). Neuroptera International 5, 189–204.

Monserrat, V.J. & Martinez, M.D. 1995. On the possible myrmecophily of Nemopterinae larvae (Neuroptera, Nemopteridae). Sociobiology 26, 55–68.

Nakahara, W. 1954. Early stages of some Japanese Hemerobiidae including two new species. Kontyû 21(1/2), 41–51.

Navás, L. 1919. Once Neurópteros nuevos españoles. Boletín de la Sociedad Entomologica de España 2, 48–56.

New, T.R. 1983. Some early stages of *Osmylops* (Neuroptera: Nymphidae). Systematic Entomology 8(1), 121–126.

Nicoli Aldini, R. 2007. Observations on the larval morphology of the antlion *Myrmeleon bore* (Tjeder, 1941) (Neuroptera Myrmeleontidae) and its life cycle in the Po Valley (northern Italy). Annali del Museo Civico di Storia Naturale di Ferrara 8, 59–66.

Parker, F.D. & Stange, L.A. 1965. Systematic and biological notes on the tribe Platymantispini (Neuroptera: Mantispidae) and the description of a new species of *Plega* from Mexico. The Canadian Entomologist 97, 604–612.

Pérez-de la Fuente, R., Engel, M.S., Delclòs, X. & Peñalver, E. 2020. Straight-jawed lacewing larvae (Neuroptera) from Lower Cretaceous Spanish amber, with an account on the known amber diversity of neuropterid immatures. Cretaceous Research 106, 104200.

Peterson, A. 1951. Larvae of Insects. An Introduction to Nearctic Species. Part II. Coleoptera, Diptera, Neuroptera, Siphonaptera, Mecoptera, Trichoptera. Edward Brothers, Columbus, OH, USA; 416 pp.

Pierre, F. 1952. Morphologie, milieu biologique et comporte­ ment de trois Crocini nouveaux du Sahara nord­occidental (Planipennes, Nemopteridae). Annales de la Société entomologique de France 119, 1–22.

Poivre, C. 1976. Observations sur la biologie, le comportement et le phénomène de convergence chez les Mantispidés [Planipennes]. Entomologiste 32, 2–19.

Riek, E.F. 1970. Neuroptera (Lacewings). In Waterhouse, D.F. (ed.), The Insects of Australia. Melbourne University Press, Melbourne, Australia, pp. 472–494.

Rojht, H., Budija, F. & Trdan, S. 2009. Effect of temperature on cannibalism rate between green lacewings larvae (*Chrysoperla carnea* [Stephens], Neuroptera, Chrysopidae). Acta Agriculturae Slovenica 93(1), 5–9.

Satar, A., Suludere, Z., Canbulat, S.A.V.A.Ş, & Oezbay, C. 2006. Rearing the larval stages of *Distoleon tetragrammicus* (Fabricius, 1798) (Neuroptera, Myrmeleontidae) from egg to adult, with notes on their behaviour. Zootaxa 1371(1), 57–64.

Satar, A., Suludere, Z., Candan, D. & Canbulat, S. 2007. Morphology and surface structure of eggs and first instar larvae of *Croce schmidti* (Navás, 1927) (Neuroptera: Nemopteridae). Zootaxa 1554, 49–55.

Stitz, H. 1931. Planipennia. In: Schultze, P. (ed.), Biologie der Tiere Deutschlands. Lfg. 33, Teil 35; Borntraeger, Berlin, Germany,

pp. 67–304.

Suludere, Z., Satar, A., Candan, S. & Canbulat, S. 2006. Morphology and surface structure of eggs and first instar larvae of *Dielocroce baudii* (Neuroptera: Nemopteridae) from Turkey. Entomological News 117, 521–530.

Tillyard, R.J. 1916. Studies in Australian Neuroptera. No. iv. The families Ithonidae, Hemerobiidae, Sisyridae, Berothidae, and the new family Trichomatidae; with a discussion of their characters and relationships, and descriptions of new and little-known genera and species. Proceedings of the Linnean Society of New South Wales 41, 269–332.

Tillyard, R.J. 1922. The life-history of the Australian moth-lacewing, *Ithone fusca*, Newman (Order Neuroptera Planipennia). Bulletin of Entomological Research 13, 205–223.

Tillyard, R.J. 1926. Order Neuroptera (Alderflies, Lacewings). In: The Insects of Australia and New Zealand, 1st ed.; Angus and Robertson, Sydney, Australia, pp. 308–325.

Toschi, C.A. 1964. Observations on *Lomamyia latipennis*, with a description of the first instar larva. Pan-Pacific Entomologist 40, 21–26.

Tusun, S. & Satar, A. 2016. Morphology, surface structure and sensory receptors of larvae of *Dielocroce ephemera* (Gerstaecker, 1894) (Neuroptera: Nemopteridae). Entomological News 126, 144–149.

Wang, B., Xia, F., Engel, M.S., Perrichot, V., Shi, G., Zhang, H., Chen, J., Jarzembowski, E.A., Wappler, T. & Rust, J. 2016. Debris-carrying camouflage among diverse lineages of Cretaceous insects. Science Advances 2(6), e1501918.

Withycombe, C.L. 1925. XV. Some aspects of the biology and morphology of the Neuroptera. With special reference to the immature stages and their possible phylogenetic significance. Transactions of the Royal Entomological Society of London 72(3–4), 303–411.
