# Supplementary material for: 100 Million-year-old straight-jawed lacewing larvae with enormously inflated trunks represent the oldest cases of extreme physogastry in insects
Source: Sci Rep. 2022 Jul 26;12:12760. doi: 10.1038/s41598-022-16698-y (PMC9325756; doi:10.1038/s41598-022-16698-y)
Supplement: Supplementary file 7 — Supplementary Information 6. [file 41598_2022_16698_MOESM7_ESM.pdf]

[Analysis Information]

Results File : \\Mac\Home\Desktop\SHAPE_physo\shape_physo.pcr
Analyzed NEF File : \\Mac\Home\Desktop\SHAPE_physo\shape_physo.nef
Title of NEF File Data :
Analyzed Date : Tue Mar 22 11:37:51 2022
Method of Analysis : Covariance
Number of Data : 50
Number of Harmonics : 20
Number of Analyzed Harmonics : 20
Constant Coefficient(s) : a1 b1 c1
Number of Principal Components : 57
(= Number of Analyzed Coefficients (NAC))
Number of Effective Principal Components : 2
(= Number of Principal Components whose proportion is larger than 1 / NAC)
Analyzed Coefficients : a2 b2 c2 a3 b3 c3 a4 b4 c4 a5 b5 c5 a6 b6 c6 a7 b7 c7 a8 b8 c8 a9 b9 c9 a10 b10 c10 a11 b11 c11 a12 b12 c12 a13 b13 c13 a14 b14 c14 a15 b15 c15 a16 b16 c16 a17 b17 c17 a18 b18 c18 a19 b19 c19 a20 b20 c20

[Mean of Coefficients]

	a	b	c	d
1	1,000000E+000	1,194582E-015	8,235123E-019	4,582083E-001
2	7,101950E-001	-2,042872E-004	-5,826506E-005	2,026791E-001
3	-2,371427E-001	2,586123E-004	1,061693E-004	1,391907E-001
4	1,704922E-001	-1,342872E-004	-7,603836E-005	6,573604E-003
5	1,634773E-002	4,648375E-005	2,375027E-006	3,828527E-002
6	-2,465426E-002	4,094079E-005	-1,202741E-005	-1,302278E-002
7	6,334195E-003	1,390689E-005	1,782339E-005	1,805412E-002
8	1,947622E-002	-2,095971E-005	2,757951E-006	4,420771E-003
9	-2,061861E-002	6,054052E-005	-1,308510E-006	6,100854E-003
10	1,046047E-002	-4,235797E-005	-1,056621E-005	-5,202083E-003
11	2,826024E-003	2,956810E-005	6,357774E-006	6,263312E-003
12	-7,347406E-003	4,287175E-005	2,795252E-006	-6,820533E-003
13	1,801841E-003	-1,350295E-005	2,229894E-006	3,373657E-003
14	2,549464E-003	1,763016E-005	1,969600E-006	-2,700135E-003
15	-2,916086E-003	7,123176E-006	2,389234E-005	2,308812E-003
16	-9,997983E-004	7,105484E-006	-2,382473E-005	-2,444607E-003
17	4,040477E-003	-9,962346E-006	-6,408789E-006	3,082523E-003
18	-3,253775E-003	4,072138E-005	-2,812018E-005	-2,495061E-003
19	1,626921E-003	-2,011677E-005	-1,236349E-005	1,145001E-003
20	4,872104E-004	6,818948E-006	2,601402E-005	-9,834745E-004

[Std of Coefficients]

	a	b	c	d
1	Constant	Constant	Constant	1,038716E-001
2	1,942932E-001	1,569595E-003	4,236196E-004	4,559689E-002
3	4,750985E-002	1,225757E-003	1,370119E-003	4,278151E-002
4	2,509720E-002	7,562746E-004	4,430202E-004	1,930239E-002
5	4,959876E-002	3,325138E-004	2,699296E-004	2,046997E-002
6	2,036294E-002	3,632547E-004	2,459505E-004	1,396556E-002
7	1,274062E-002	2,617940E-004	3,054459E-004	1,149163E-002
8	1,967413E-002	3,321254E-004	1,818351E-004	9,525882E-003
9	1,399283E-002	3,430388E-004	2,017524E-004	9,918676E-003
10	1,575727E-002	2,659182E-004	2,538363E-004	7,664595E-003
11	1,766838E-002	1,840190E-004	1,250362E-004	7,016882E-003
12	1,213142E-002	2,738290E-004	1,709527E-004	5,689685E-003
13	7,928564E-003	1,818800E-004	1,439203E-004	5,465038E-003
14	6,123614E-003	1,575246E-004	1,257763E-004	3,957312E-003
15	6,540827E-003	2,389844E-004	1,324383E-004	3,181850E-003
16	7,920741E-003	1,839692E-004	1,379388E-004	2,698337E-003
17	7,977541E-003	1,475064E-004	9,885737E-005	2,960447E-003
18	6,106864E-003	1,873049E-004	1,402893E-004	3,144934E-003
19	4,987923E-003	1,348429E-004	1,172093E-004	2,934483E-003
20	3,209492E-003	1,058434E-004	1,109253E-004	2,679098E-003

[Eigenvalue and Proportion]

	Eigenvalue	Proportion(%)	Cumulative(%)	> 1/57
Prin1	4,321180E-002	95,3593	95,3593	*
Prin2	9,247290E-004	2,0407	97,4000	*
Prin3	4,628476E-004	1,0214	98,4214	
Prin4	3,195390E-004	0,7052	99,1266	
Prin5	1,337978E-004	0,2953	99,4218	
Prin6	7,238833E-005	0,1597	99,5816	

Prin7	5,710907E-005	0,1260	99,7076
Prin8	4,717550E-005	0,1041	99,8117
Prin9	3,237485E-005	0,0714	99,8831
Prin10	1,453146E-005	0,0321	99,9152
Prin11	1,164472E-005	0,0257	99,9409
Prin12	8,400458E-006	0,0185	99,9595
Prin13	5,122868E-006	0,0113	99,9708
Prin14	3,849581E-006	0,0085	99,9793
Prin15	2,484079E-006	0,0055	99,9847
Prin16	2,039357E-006	0,0045	99,9892
Prin17	1,380830E-006	0,0030	99,9923
Prin18	1,027132E-006	0,0023	99,9945
Prin19	7,064481E-007	0,0016	99,9961
Prin20	4,729375E-007	0,0010	99,9972
Prin21	4,112181E-007	0,0009	99,9981
Prin22	3,261973E-007	0,0007	99,9988
Prin23	2,284278E-007	0,0005	99,9993
Prin24	1,129138E-007	0,0002	99,9995
Prin25	6,929970E-008	0,0002	99,9997
Prin26	3,240160E-008	0,0001	99,9998
Prin27	2,480711E-008	0,0001	99,9998
Prin28	1,701028E-008	0,0000	99,9998
Prin29	1,589115E-008	0,0000	99,9999
Prin30	1,225682E-008	0,0000	99,9999
Prin31	1,107166E-008	0,0000	99,9999
Prin32	8,482317E-009	0,0000	100,0000
Prin33	5,198688E-009	0,0000	100,0000
Prin34	4,084406E-009	0,0000	100,0000
Prin35	2,589710E-009	0,0000	100,0000
Prin36	2,320021E-009	0,0000	100,0000
Prin37	1,883286E-009	0,0000	100,0000
Prin38	1,538820E-009	0,0000	100,0000
Prin39	8,151156E-010	0,0000	100,0000
Prin40	5,820950E-010	0,0000	100,0000
Prin41	4,398678E-010	0,0000	100,0000
Prin42	3,447466E-010	0,0000	100,0000
Prin43	2,384088E-010	0,0000	100,0000
Prin44	1,716661E-010	0,0000	100,0000
Prin45	1,031927E-010	0,0000	100,0000
Prin46	5,352198E-011	0,0000	100,0000
Prin47	3,564549E-011	0,0000	100,0000
Prin48	1,752461E-011	0,0000	100,0000
Prin49	5,159277E-012	0,0000	100,0000
Prin50	2,686660E-022	0,0000	100,0000
Prin51	7,783185E-023	0,0000	100,0000
Prin52	-2,908857E-024	0,0000	100,0000
Prin53	-2,887505E-023	0,0000	100,0000
Prin54	-4,328720E-023	0,0000	100,0000
Prin55	-1,828834E-022	0,0000	100,0000
Prin56	-4,035947E-022	0,0000	100,0000
Prin57	-3,546709E-021	0,0000	100,0000

Total Variance : 4,531471E-002
